# Tight focusing of fractional-order topological charge vector beams by cascading metamaterials and metalens

**DOI:** 10.1038/s41378-024-00781-7

**Published:** 2024-10-16

**Authors:** Lan Ke, Yunyun Yang, Mingmin Zhu, Haomiao Zhou, Yi Chen, Ying Tian, Chenxia Li, Bo Fang, Zhi Hong, Xufeng Jing

**Affiliations:** 1 The Institute of Optoelectronic Technology, China Jiliang University, Hangzhou, China; 2 The College of Standardization, China Jiliang University, Hangzhou, PR China; 3 The College of Information Engineering, China Jiliang University, Hangzhou, China; 4 College of Metrology & Measurement Engineering, China Jiliang University, Hangzhou, China; 5 Centre for THz Research, China Jiliang University, Hangzhou, China

**Keywords:** Optical physics, Optical physics

## Abstract

Vector beams have attracted widespread attention because of their unique optical properties; in particular, their combination with tight focusing can produce many interesting phenomena. The rise of 3D printing technology provides more possibilities for exploration. In this work, a cascading method involving a metamaterial and a metalens is used to generate a tightly focused field of vector beams in the terahertz band, which is prepared via 3D printing. As a proof-of-concept demonstration, a series of metamaterial modules capable of generating states of different orbital angular momentum are proposed by cascading with a metalens. The experimental results are in good agreement with the simulation results, fully verifying the feasibility of the scheme. The proposed design and fabrication strategy provides a new idea for the tight focusing of terahertz vector beams.

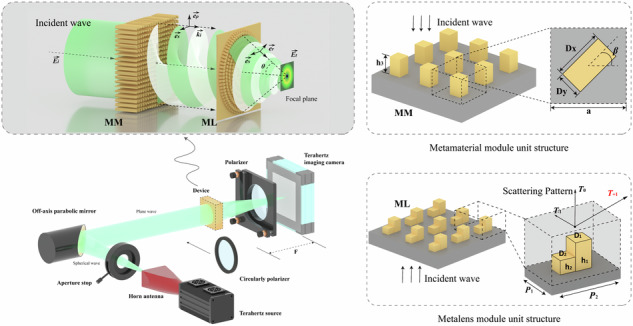

## Introduction

Electromagnetic waves carrying orbital angular momentum (OAM), known as vortex beams, have a helical phase exp(i*lφ*)^1,2^. *l* is the topological charge of the vortex beam and the number of 2π phase periods around the central singularity, and each photon in the beam carries *l*ℏ of OAM^3,4^. Vortex beams with different topological charges play a very important role in high-capacity communication because their arbitrary modes are orthogonal to each other and are widely used in particle capture^5,6^, information transmission^7^, and superresolution imaging^8^. Since *l* can have high degrees of freedom values, the fractional topological charge of the vortex beam can increase its encoding ability, produce a discontinuous spiral phase and cause changes in the light intensity distribution, which results in a wider range of development prospects for the vortex beam^9^.

In addition to phase properties, polarization is also an important property of light beams and plays an important role in the interaction between light and matter^10^. In the past, researchers studied spatially uniform polarization states. These polarization states have nothing to do with the spatial position of the beam cross section. In recent years, the propagation and focusing properties of light beams with nonuniform distributions of polarization space have received considerable attention^11–14^, which can be applied to information storage^15^, optical communication^16^, and other fields^10^.

Combining beams with different vector properties with high numerical aperture lenses can produce unique tight focusing performance, disrupting the focusing properties under traditional scalar diffraction theory^17^. Spatial light modulators^18^, axial birefringent elements^19,20^, and metamaterials^21–24^ can all be used to generate vector beams. In the terahertz wave band, the traditional equipment required to generate the vector tight focusing field is complex, numerous and expensive^25–30^, which is very unfavorable for actual research on the terahertz vector beam tight focusing field^31–34^. Therefore, we chose to use metamaterials^35–38^ and a metalens^22,32,34,39–41^ to generate and focus different vector beams.

Metamaterials are artificial materials with special properties that can effectively and flexibly control electromagnetic waves, and metasurfaces are two-dimensional manifestations of metamaterials^37,42–46^. Combining the two makes it possible to adjust electromagnetic waves more carefully while modulating their polarization characteristics, amplitude, and phase^47–53^.

We have previously successfully achieved tight focusing of cylindrical vector beams via cascaded metamaterials. However, the previous work was limited to two special cases of cylindrical vector beams: radial and azimuthal vector beams. These two types of beams have relatively simple vector characteristics and tight focusing properties and are limited to the study of integer-order vector beams. To conduct a more in-depth analysis of vector beams, we further extend the study of vector beams to include fractional-order vortex beams and tight focusing. In addition to integer-order vortex beams, we further analyze the tight focusing state of fractional-order vortex beams. The asymmetric focal field distribution caused by fractional order can result in more interesting performance and applications, such as particle manipulation and sorting, or provide new degrees of freedom for applications such as all-optical magnetic recording. Under the tight focusing state, we first compare the performance differences between integer-order vortex beams and fractional-order vortex beams with different topological charges. Next, we delve into the different vortex beam characteristic distributions under the superposition of conjugate topological charges.

Exploring various scenarios increases the difficulty of preparing the metasurface. 3D printing technology can effectively solve this problem. Previous studies have demonstrated that 3D printing technology is a flexible and cost-effective fabrication method. In combination with 3D printing technology, and utilizing low-refractive-index materials for construction, we can freely study tight focusing under various states with almost no time cost for preparation. Therefore, in this work, we designed multiple metamaterial modules and a metalens module using the same material. The metamaterial module can generate beams with different vector characteristics. When physically cascaded with the metalens module, multiple tightly focused fields are generated under different polarization states of the incident light mode. The focusing characteristics in different modes are studied via numerical simulation. On the basis of 3D printing technology, we prepared the designed module and built a test optical path for experimental verification. The simulation results and experimental results prove the feasibility and flexibility of the proposed scheme, including the tight focusing characteristics of integer-order vortex beams, fractional-order vortex beams, and OAM superposition mode vector beams.

## Design principle

To research the tightly focused field of the vortex beam with different topological charges and the superimposed vector beam of the conjugate topological charge, we separately design the vector beam generating part and the lens part. We can ensure the flatness of the docking part of the two modules and then use the planar characteristics of the two modules for cascading.

**Fig. 1:**
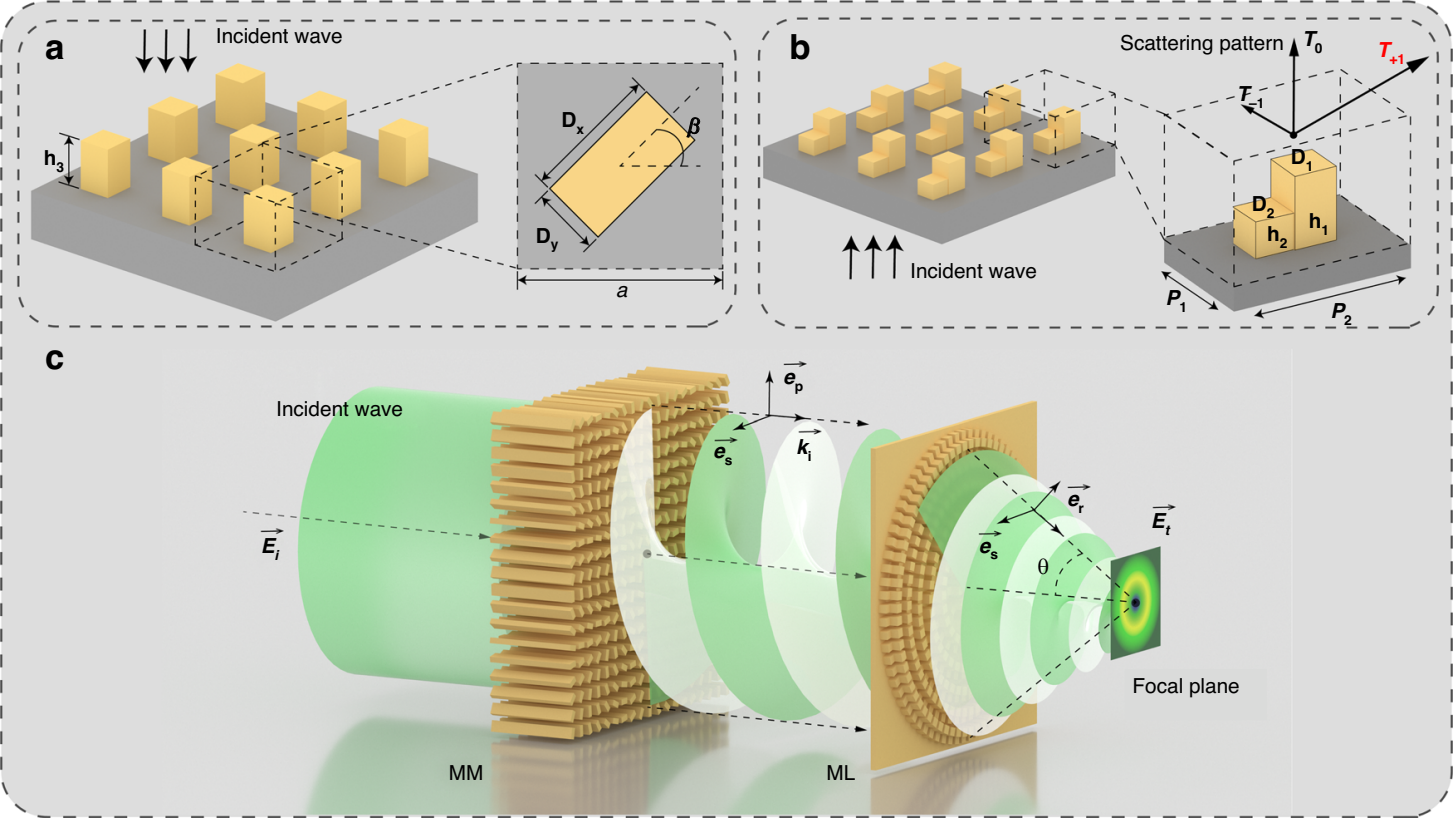
Cascading metamaterials generate vector beams with tightly focused fields. **a** Perspective schematic diagram and top view of the metamaterial module unit structure. **b** Perspective schematic diagram of the structure of the metalens module unit and its scattering mode. **c** Schematic diagram of beam conversion by cascade modules

The metamaterial module (MM) is composed of rectangular column units, and the unit structure is shown in Fig. 1a. Each rectangular column can independently change the major axis length *D*_*x*_, the minor axis length *D*_*y*_, the height h_3_, and the rotation angle *β* to adjust the polarization and phase of the incident light. The columns are spaced at intervals of a period *a*. A rectangular column unit will produce an effect similar to that of the F-P resonator to confine the incident light^31^. Considering the incident beam as a superposition of two orthogonal linear polarizations, the change in *D*_*x*_ causes the column structure to introduce an additional phase factor exp(i*φ*_*x*_) for the x-polarized component of the incident beam^54–57^. Similarly, changing *D*_*y*_ introduces an additional phase factor exp(i*φ*_*y*_). An increase in the height gradually increases the phase difference accumulated between the two additional phases during the transmission of the beam. Finally, the rotation angle *β* is introduced, and the transformation effect of each unit on the beam can be expressed by the Jones matrix^58^:

1 $$T=\left[\begin{array}{cc}\cos \beta & -\,\sin \beta \\ \sin \beta & \cos \beta \end{array}\right]\left[\begin{array}{cc}{e}^{i{\varphi }_{x}} & 0\\ 0 & {e}^{i{\varphi }_{y}}\end{array}\right]\left[\begin{array}{cc}\cos \beta & \sin \beta \\ -\,\sin \beta & \cos \beta \end{array}\right]$$

When we assign the properties of a half-wave plate to a rectangular column, that is, exp(i*φ*_*x*_) = 1, exp(i*φ*_*y*_) = −1, each unit has the following effects on an incident left-handed circularly polarized (LHCP) beam:

2 $$\begin{array}{c}{E}_{out}={E}_{in}\times T={E}_{0}\left[\begin{array}{c}1\\ i\end{array}\right]\exp i{\varphi }_{0}\times \left[\begin{array}{cc}\cos 2\beta & \sin 2\beta \\ \sin 2\beta & -\,\cos 2\beta \end{array}\right]\\ ={E}_{0}\left[\begin{array}{c}1\\ -i\end{array}\right]\exp \left[i(2\beta +{\varphi }_{0})\right]\end{array}$$

where *E*_*0*_ is the amplitude and *φ*_*0*_ is the initial phase. A vector beam with vortex phase exp(i*φ*) can be expressed with Eq. (3) as:

3 $$|{\rm{LHCP}}\rangle ={E}_{0}\left[\begin{array}{c}1\\ -i\end{array}\right]\exp \left[i(l\varphi +{\varphi }_{0})\right]$$

*φ* is the azimuth angle, so we can obtain the relationship between the rotation angle of the metamaterial unit and the azimuth angle when designing vortex beams with different topological charges:

4 $$\varphi =\frac{2\beta }{l}$$

After the unit size is determined, the parameter adjustment for constructing the array is simplified to a single adjustment of *β*. Right-handed circularly polarized (RHCP) light can be obtained in the same way:

5 $$|{\rm{RHCP}}\rangle ={E}_{0}\left[\begin{array}{c}1\\ i\end{array}\right]\exp [-i(2\beta +{\varphi }_{0})]$$

For the same rotation angle, the incidence of orthogonal circularly polarized light will generate vortex light with conjugate topological charges. When we superpose a pair of vortex beams with conjugate topological charges, we can obtain:

6 $$\begin{array}{c}{E}_{vector}=|{\rm{LHCP}}\rangle +|{\rm{RHCP}}\rangle =\frac{1}{2}{E}_{0}\left[\begin{array}{c}1\\ -i\end{array}\right]\exp \left[i(l\varphi +{\varphi }_{0})\right]+\frac{1}{2}{E}_{0}\left[\begin{array}{c}1\\ i\end{array}\right]\exp \left[-i(l\varphi +{\varphi }_{0})\right]\\ ={E}_{0}\left[\begin{array}{c}\frac{1}{2}\exp \left[i(l\varphi +{\varphi }_{0})\right]+\frac{1}{2}\exp \left[-i(l\varphi +{\varphi }_{0})\right]\\ \frac{1}{2}\exp \left[i(l\varphi +{\varphi }_{0})\right]-\frac{1}{2}\exp \left[-i(l\varphi +{\varphi }_{0})\right]\end{array}\right]={E}_{0}\left[\begin{array}{c}\cos (l\varphi +{\varphi }_{0})\\ \sin (l\varphi +{\varphi }_{0})\end{array}\right]\end{array}$$

Equation (6) shows that the superposition of these two vortex beams with orthogonal polarization and conjugate topological charge generates a cylindrical vector beam with *l*-order polarization distribution, and the simultaneous incidence of a pair of orthogonal circular polarizations of the same size can be equivalent to linear polarization incidence. By changing the distribution of metamaterial units, we designed four arrays corresponding to different topological charges. The first are two modules that can generate beams with *l* = 5 and *l* = 9. On the basis of these two metamaterials, *l* = 4.5 and *l* = 8.5 metamaterials were designed to explore the different characteristics of fractional vortex beams in tight focusing and conjugate superposition tight focusing. When metamaterials with different topological charges are designed, only the rotation angle of the unit needs to be changed, which simplifies the difficulty of design. The production process of 3D printing is convenient and fast, and we can conveniently prepare the required modules to research the state characteristics of tight focusing fields with different topological charges.

After the generation of the vector beam is completed, we also need a lens with a high numerical aperture to confine the generated beam. To facilitate integration with the metamaterial module, we chose the same material for design to further simplify the 3D printing preparation process, and the flat characteristic of the metasurface also ensures that we can easily cascade the two modules. The traditional phase gradient mapping mode needs to fill the 0-2π phase interval with cells with continuous phase gradients, which limits the size of the metalens^48,51,53^. The fixed unit period also makes it impossible for us to match the corresponding length of the entire phase interval in detail. In the case of a small array density, the discretized phase distribution weakens the transformation effect of the lens. Here, we designed the metalens by using the diffractive energy distribution properties of metasurface elements with asymmetric scattering modes^59^. As shown in Fig. 1b, the unit is constructed of asymmetric square pillars. Two square pillars of different sizes and heights are built on the rectangular unit substrate. The side lengths of the two pillars are *D*_*1*_ and *D*_*2*_, and the heights are *h*_*1*_ and *h*_*2*_. This asymmetric structure can concentrate the diffraction energy on the T_+1_ order, realize the redistribution of the diffraction energy, and deflect the outgoing beam with a single unit. Moreover, the thickness of the unit is much smaller than that of the metalens module, which reduces the size of the cascaded device. The angle of beam deflection is regulated by the diffraction period *P*_*2*_ of the unit, and the specific calculation is in accordance with the following equation:

7 $$\delta ={\sin }^{-1}(\frac{\lambda }{{P}_{2}})$$

where *δ* is the diffraction angle and *λ* is the wavelength. For a given wavelength and diffraction angle, we can determine the size of *P*_*2*_, and the short side length *P*_*1*_ is the nondiffraction period, which does not affect the diffraction. Therefore, the value of *P*_*1*_ should be as small as possible to avoid diffraction while maintaining a high array density. The numerical aperture of our designed metalens is 0.87, the focal length *F* is 16 mm, and its maximum scattering angle is 60°. The size of the lens is limited by the focal length, and cells on different regions of the lens are designed to produce different deflection angles corresponding to their radial position within the lens, thereby approximating the ideal parabolic phase map to a piecewise linear phase map. The nondiffractive period is held constant, whereas the diffractive period is varied to produce the first diffractive order at the corresponding angle. The unit structure on each ring belt is integrated as much as possible under the premise of ensuring the deflection effect to maximize the energy conversion efficiency.

The two designed modules are physically cascaded, as shown in Fig. 1c. The metamaterial module MM can convert incident light of different polarization states, owing to the vector characteristics required by the experiment. The vector beam converted by the MM is subsequently collected by a cascaded high numerical aperture metalens module (ML) to generate a tightly focused spot at the focal plane. The output beam transformed by cascaded devices is represented as:

8 $${{\boldsymbol{E}}}_{{\boldsymbol{t}}}(\theta ,\varphi )=({{\boldsymbol{E}}}_{{\boldsymbol{i}}}\cdot {{\boldsymbol{e}}}_{{\boldsymbol{p}}})\cdot {{\boldsymbol{e}}}_{{\boldsymbol{r}}}+({{\boldsymbol{E}}}_{{\boldsymbol{i}}}\cdot {{\boldsymbol{e}}}_{{\boldsymbol{s}}})\cdot {{\boldsymbol{e}}}_{{\boldsymbol{s}}}$$

among them:

9 $${{\boldsymbol{e}}}_{{\boldsymbol{p}}}=\,\cos \varphi {\boldsymbol{i}}+\,\sin \varphi {\boldsymbol{j}}=\left[\begin{array}{c}\cos \varphi \\ \sin \varphi \\ 0\end{array}\right]$$

10 $${{\boldsymbol{e}}}_{{\boldsymbol{s}}}={{\boldsymbol{k}}}_{{\boldsymbol{i}}}\times {{\boldsymbol{e}}}_{{\boldsymbol{p}}}=-\sin \varphi {\boldsymbol{i}}+\,\cos \varphi {\boldsymbol{j}}=\left[\begin{array}{c}-\,\sin \varphi \\ \cos \varphi \\ 0\end{array}\right]$$

11 $${{\boldsymbol{e}}}_{{\boldsymbol{r}}}=\,\cos \theta (\cos \varphi {\boldsymbol{i}}+\,\sin \varphi {\boldsymbol{j}})+\,\sin \theta {\boldsymbol{k}}=\left[\begin{array}{c}\cos \theta \,\cos \varphi \\ \cos \theta \,\sin \varphi \\ -\,\sin \theta \end{array}\right]$$

where ***k***_***i***_ is the wave vector and ***e***_***ρ***_ and ***e***_***s***_ are the radial and angular components, respectively, *e*_*r*_ is the radial component after lens conversion, and *θ* is the maximum deflection angle of the lens. Therefore, the output field of left-handed circularly polarized light and right-handed circularly polarized light can be expressed as:

12 $$\begin{array}{l}{{\boldsymbol{E}}}_{{\boldsymbol{t}}{\boldsymbol{L}}}=\frac{1}{\sqrt{2}}{e}^{i({l}_{L}+1)\varphi}\left[(\cos \varphi \,\cos \theta -i\,\sin \varphi )\cdot {\boldsymbol{i}}\right.\\ \qquad\qquad+\,(\sin \varphi \,\cos \theta +i\,\cos \varphi )\cdot {\boldsymbol{j}}-\,\sin \theta \cdot {\boldsymbol{k}}\left]\right.\end{array}$$

13 $$\begin{array}{c}{{\boldsymbol{E}}}_{{\boldsymbol{t}}{\boldsymbol{R}}}=\frac{1}{\sqrt{2}}{e}^{i({l}_{R}-1)\varphi }\left[(\cos \varphi \,\cos \theta +i\,\sin \varphi )\cdot {\boldsymbol{i}}\right.\\ \qquad\qquad+\,(\sin \varphi \,\cos \theta -i\,\cos \varphi )\cdot {\boldsymbol{j}}-\,\sin \theta \cdot {\boldsymbol{k}}\left]\right.\end{array}$$

We can derive the field distribution after focusing on the Richard Wolf vector diffraction integral^60^, which is as follows:

14 $${{\boldsymbol{E}}}_{{\boldsymbol{s}}}=\frac{-ik}{2\pi }{\iint }_{\varOmega }{\boldsymbol{a}}(\theta ,\varphi )\exp [ik(z\,\cos \theta +\rho \,\sin \theta \,\cos (\varphi -{\varphi }_{s})]d\varOmega$$

where ${\bf{a}}(\theta ,\varphi )=\sqrt{\cos \theta }{{\boldsymbol{E}}}_{{\boldsymbol{t}}}(\theta ,\varphi )$ is the weight function of the electric field. The integration is simplified by the first kind of n-order Bessel function with left-handed circularly polarized light incidence as:

15 $$\begin{array}{c}{\int_{0}^{2\pi }}\exp (in\varphi )\exp [ik\rho \,\sin \theta \,\cos (\varphi -{\varphi }_{s})]d\varphi \\ =2\pi {i}^{n}{J}_{n}(k\rho \,\sin \theta )\exp (in{\Phi })\end{array}$$

**Fig. 2:**
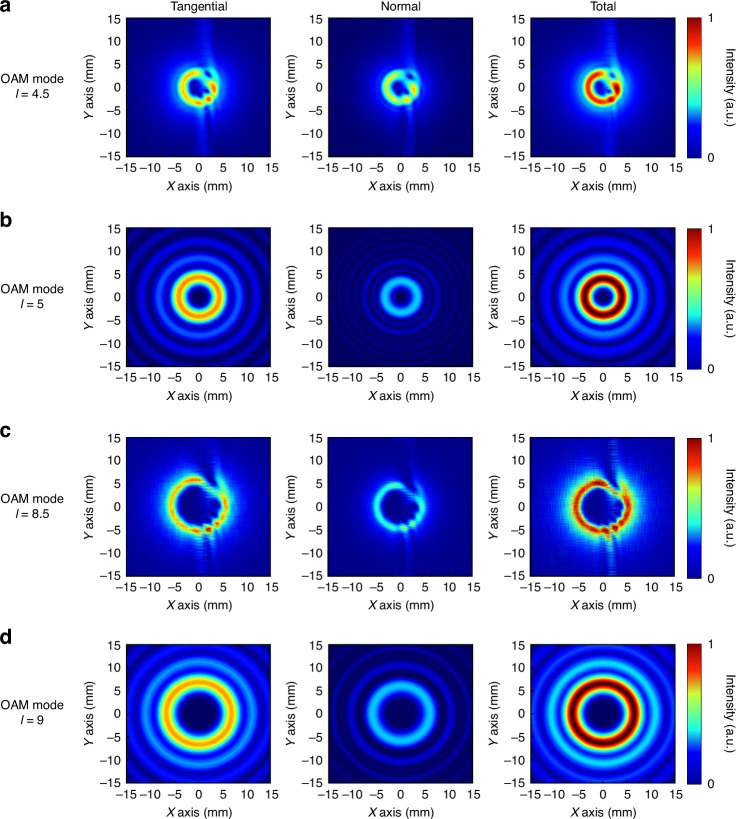
Analysis of a tightly focused field with left-handed circularly polarized incident light. **a** Focal plane sampling analysis of the *l* = 4.5 mode. **b** Focal plane sampling analysis of the *l* = 5 mode. **c** Focal plane sampling analysis of the *l* = 8.5 mode. **d** Focal plane sampling analysis of the *l* = 9 mode

**Fig. 3:**
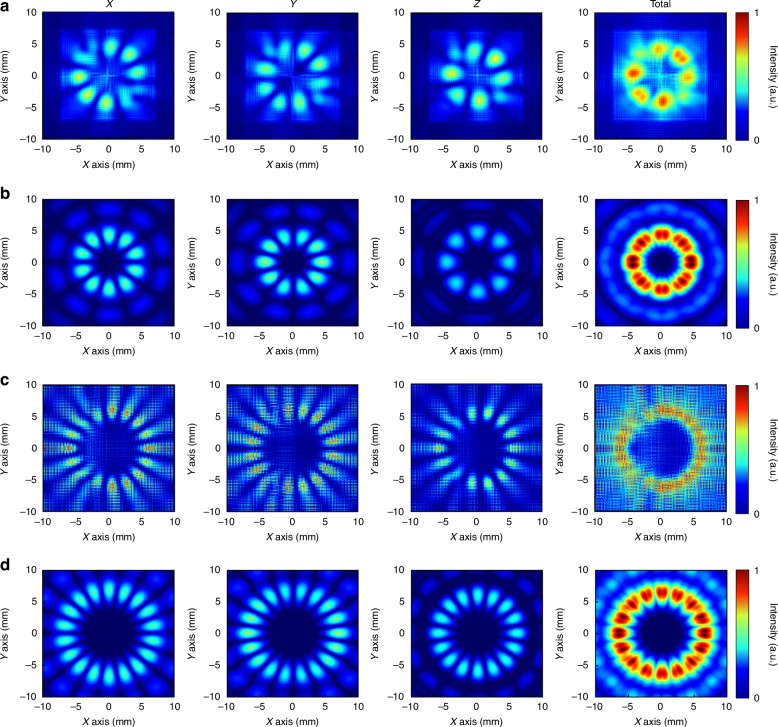
Analysis of a tightly focused field with conjugate OAM superimposed vector beams. **a** Focal plane sampling analysis of the *l* = 4.5 mode. **b** Focal plane sampling analysis of the *l* = 5 mode. **c** Focal plane sampling analysis of the *l* = 8.5 mode. **d** Focal plane sampling analysis of the *l* = 9 mode

We ultimately obtain three component expressions in the spatial coordinate system of the focusing field:

16 $$\begin{array}{ll}{{\boldsymbol{E}}}_{{\boldsymbol{x}}{\boldsymbol{L}}}=\pi {\int_{0}^{\alpha }}\sqrt{\cos \theta }\,\sin \theta \exp (ikz\,\cos \theta )\\ \qquad\quad\times\, \{(1-\,\cos \theta )[{i}^{l}A\exp (i\varphi l+2i\varphi )]+(1+\,\cos \theta )[{i}^{l}B\exp (i\varphi l)]\}d\theta \end{array}$$

17 $$\begin{array}{ll}{{\boldsymbol{E}}}_{{\boldsymbol{y}}{\boldsymbol{L}}}=-i\pi {\int_{0}^{\alpha }}\sqrt{\cos \theta }\,\sin \theta \exp (ikz\,\cos \theta )\\ \qquad\qquad\times \,\{(1-\,\cos \theta )[{i}^{l}A\exp (i\varphi l+2i\varphi )]-(1+\,\cos \theta )[{i}^{l}B\exp (i\varphi l)]\}d\theta \end{array}$$

18 $$\begin{array}{ll}{{\boldsymbol{E}}}_{{\boldsymbol{z}}{\boldsymbol{L}}}=-{\int_{0}^{\alpha }}\sqrt{\cos \theta }{\sin }^{2}\theta \exp (ikz\,\cos \theta )\\ \qquad\qquad\times \,[2\pi {i}^{(l-1)}{J}_{(l-1)}(k\rho \,\sin \theta )\exp (i\varphi l-i\varphi )]d\theta \end{array}$$

where *A* = *J*_(*l*+2)_(*kρ*sin*θ*) and *B* = *J*_*l*_(*kρ*sin*θ*). Similarly, the incident case of right-handed circular polarization can be obtained as:

19 $$\begin{array}{l}{{\boldsymbol{E}}}_{{\boldsymbol{x}}{\boldsymbol{R}}}=\pi {\int_{0}^{\alpha }}\sqrt{\cos \theta }\,\sin \theta \exp (ikz\,\cos \theta )\\ \qquad\quad\times\, \{(1-\,\cos \theta )[{i}^{-l}C\exp (i\varphi ({-}l)-2i\varphi )]+(1+\,\cos \theta )[{i}^{-l}D\exp (i\varphi ({-}l))]\}d\theta \end{array}$$

20 $$\begin{array}{ll}{{\boldsymbol{E}}}_{{\boldsymbol{y}}{\boldsymbol{R}}}=i\pi {\int_{0}^{\alpha }}\sqrt{\cos \theta }\,\sin \theta \exp (ikz\,\cos \theta )\\ \qquad\quad\times\, \{(1-\,\cos \theta )[{i}^{{l}_{R}}C\exp (i\varphi (-l)-2i\varphi )]+(1+\,\cos \theta )[{i}^{-l}D\exp (i\varphi (-l))]\}d\theta \end{array}$$

21 $$\begin{array}{ll}{{\boldsymbol{E}}}_{{\boldsymbol{z}}{\boldsymbol{R}}}=-{\int_{0}^{\alpha }}\sqrt{\cos \theta }{\sin }^{2}\theta \exp (ikz\,\cos \theta )\\ \qquad\quad\times \,[2\pi {i}^{(1-l)}{J}_{(1-l)}(k\rho \,\sin \theta )\exp (i\varphi (-l)+i\varphi )]d\theta \end{array}$$

where *C* = *J*_(-*l*-2)_(*kρ*sin*θ*) and *D* = *J-*_*l*_(*kρ*sin*θ*). On the basis of the above equation, we can obtain the theoretically expected focusing field effect, as shown in Figs. 2 and 3. All the results are normalized.

## Results

Figure 2 shows the tightly focused field of a vortex beam obtained via Eqs. (16)–(18), where the incident beam is left-handed circularly polarized light. Owing to the close relationship between the order of the first-order Bessel function and the topological charge, when solving with fractional orders, calculations are performed via equations that have not been simplified by Bessel’s identity. The results of integer-order solutions exhibit an overall circular ring distribution, with the tangential component having a slightly larger ring radius than the normal component and a significantly higher intensity. On the other hand, fractional-order solutions exhibit a gapped ring distribution, with the intensity and ring radius showing a trend similar to that of the integer-order solutions. The results of superimposing OAM beams with conjugate topological charges are shown in Fig. 3. The superposition of integer-order beams produces a total field with a petal-shaped distribution, as shown in Fig. 3b, d, with all the x, y, and z components exhibiting a uniform multifocal point distribution. The number of focal points in the x and y components is 2*l*, whereas in the z component, it is 2*(*l*-1). The introduction of fractional orders results in two gaps in the overall intensity, as shown in Fig. 3a, c. From the x, y, and z components, polarization conflicts result in intensity blurring and cancellation, and the distribution is similar to that of integer orders.

We utilize 3D printing technology for sample fabrication. Conventional photolithography methods struggle to construct structures simultaneously on both sides of a material^14,61–81^. 3D printing enables the production of objects with intricate internal structures and complex geometries, achieving details that are challenging for conventional manufacturing methods. Its capabilities in customized production and rapid manufacturing greatly facilitate our experimental workflow. Compared with conventional manufacturing methods, 3D printing uses a layer-by-layer stacking approach, eliminating the need for additional tools or molds and thereby shortening the production cycle of multiple modules. Furthermore, by modifying the digital model, we can flexibly adjust the dimensions, shapes, and designs of the product to meet individual requirements, virtually eliminating any time cost associated with fabrication and other photolithography expenses from design to the final product. This is highly advantageous for our experimental research on the tight focusing of various vector beams.

**Fig. 4:**
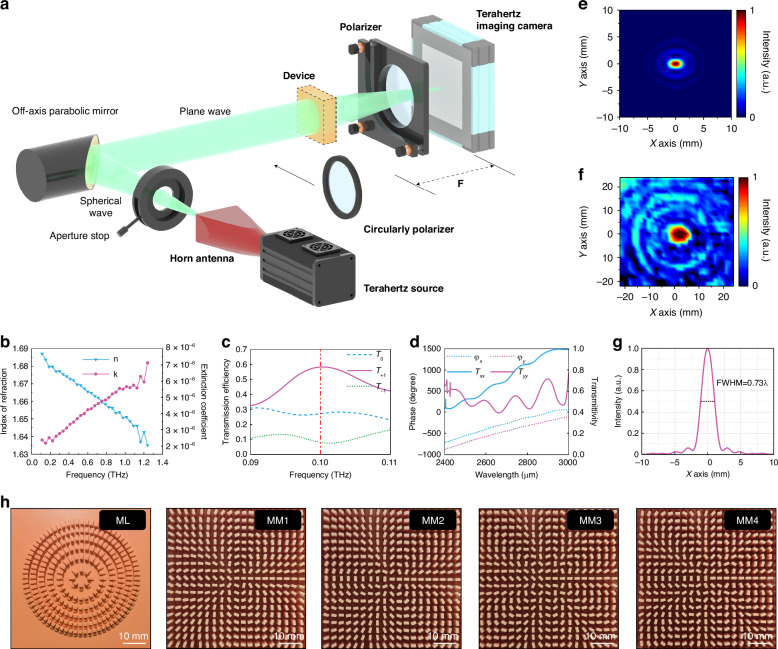
Experimental optical path, experimental test results, and sample photos. **a** Schematic diagram of the experimental light path. **b** Material properties. **c** Diffraction efficiency of the metalens unit. **d** Unit transmittance and phase distribution of the metamaterial module. **e** Normalized sampling intensity distribution of the metalens focal plane obtained via simulation. **f** The normalized sampling intensity distribution of the focal plane of the metalens obtained from the experimental test. **g** The simulated light intensity curve of the metalens module on the *x*-axis. **h** Top view of the prepared sample

The 3D printer we chose is the Shape 1+ printer of the RAYSHAPE Company, which uses low peeling force DLP light curing technology to prepare the samples. The printing pixel size is 100 μm, the printing layer thickness is 25–300 μm, and the printing tolerance is 0.05 mm, which provides us with a benchmark for unit selection. Among the materials supported by this 3D printer, we chose red wax. This resin-type material offers the best expressiveness in detail to ensure that the designed unit structure can be produced accurately and effectively. The material parameters measured by the experiment are shown in Fig. 4b. At the operating frequency (0.1 THz) we designed, the loss of the red wax material was relatively low. The subsequent numerical simulations are based on this parameter.

Through parameter scanning optimization combined with the preparation conditions, the simulation results of the diffraction efficiency of metalens asymmetric elements that we finally determined are shown in Fig. 4c. At a frequency of 0.1 THz, the *T*_+1_ order has the highest diffraction efficiency, which is much greater than those of the *T*_0_ and *T*_−1_ orders, whereas the *T*_−1_ order maintains the lowest efficiency to ensure the deflection effect. The side lengths of the two square columns are *D*_*1*_ = 1.3 mm and *D*_*2*_ = 1.4 mm, and the column heights are *h*_*1*_ = 1.5 mm and *h*_*2*_ = 3 mm. The transmittance and phase of the metamaterial module unit are shown in Fig. 4d. In a certain wavelength range, the unit can maintain the properties of a half-wave plate, reducing possible noise interference in the experiment. The period of the unit is *a* = 3 mm, the column height *h*_*3*_ = 16 mm, the minor axis length *D*_*x*_ = 1 mm, and the major axis length *D*_*y*_ = 2 mm.

On the basis of these two units, we constructed two modules. Numerical simulations and experimental verification are carried out on the designed modules. The specific experimental optical path is shown in Fig. 4a. The light source used was the terahertz source of the TeraSense Company, and the device can work in the frequency range of 0.1–0.3 THz. The terahertz light source is connected with the horn antenna (model Anteral SGH-26-WR10) to transmit the beam, and the transmission mode is a linearly polarized spherical wave. In addition to the horn antenna, the aperture diaphragm is used to perform beam shaping in the early stage, and then an off-axis parabolic mirror (manufactured by ThorLabs, model MPD369-M01) is set to perform aplanatic processing on the outgoing wave and irradiate the device we prepared. Moreover, the distance from the light source to the sample should be sufficiently large to ensure the plane wave characteristics of the incident light. Owing to the different polarization characteristics of the required incident beam, we can use a circular polarizer to convert the linearly polarized light generated by the light source into circularly polarized light. The outgoing light converted by the cascade device is received by the terahertz camera produced by TeraSense, and the results received by the terahertz camera are processed on the computer side. The model is Tera-1024. The receiving plane is composed of 32 × 32 pixels, each pixel has a side length of 1.5 mm, the working frequency band is 50 GHz–0.7 THz, and the high-speed image acquisition rate can reach 50 frames per second. We can place the terahertz camera at a distance of 16 mm from the sample and debug the overall optical path first without loading the design module to ensure the stability of the output light intensity of the light source. Moreover, the light intensity is constrained to be a circular spot slightly smaller than the camera sampling plane to facilitate the distinction of the boundary of the experimental spot. To detect different vector characteristics when linearly polarized light is incident, we place a wire grid polarizer (PW010-025-075) in front of the light source for filtering. The prepared samples were subsequently tested, and all the test results were normalized.

Different modules were fastened with a fixture when cascading, and the area required for the fixture was reserved in advance at the edge of the substrate during design.

Figure 4e shows a schematic diagram of the electric field intensity distribution at the focal plane of a separate metalens module in the linearly polarized incident mode. Sampling analysis was carried out at a focal length of 16 mm. The lens produced an elliptical focal spot with concentrated light intensity. Since the lens itself is completely symmetrically distributed, the incidence of orthogonal polarization can be kept consistent. Figure 4g shows the intensity distribution curve of the y-axis section, and its full width at half maximum (FWHM) is 0.73*λ*.

The normalized electric field intensity distribution measured by the experiment is shown in Fig. 4f. When the sample is placed 16 mm in front of the camera, the focal spot size obtained is ~6 mm, which results in an elliptical focus. The number of pixels for the actual tested spot affects the imaging results, resulting in blurring of the edges of the experimental spot. This results in a certain degree of error in the size of the tested spot, which is larger than the simulated spot. However, the overall shape of the spot matches the simulation results.

During actual sample preparation, it is not possible to maintain absolute consistency with the simulation because the manufacturing tolerances of 3D printing cause deviations in the actual dimensional parameters of the samples. Additionally, the use of red wax as a resin unavoidably results in thermal deformation, leading to a certain degree of distortion in our samples, which reduces the effectiveness of our experimental tests. Upon examination of the samples prepared in practice, the most severe deformation is observed in the outer two rings of the samples. Therefore, it is advisable to intentionally reserve redundant portions during preparation and create larger samples to ensure that the central portion of the samples can closely approximate the simulation. Therefore, we scale the array density to 25 × 25 to avoid deformation in the actual production process. Finally, we obtain four metamaterial modules for experimental testing, namely, MM1 (*l* = 4.5), MM2 (*l* = 5), MM3 (*l* = 8.5), and MM4 (*l* = 9). The physical images of all the prepared samples are shown in Fig. 4h.

**Fig. 5:**
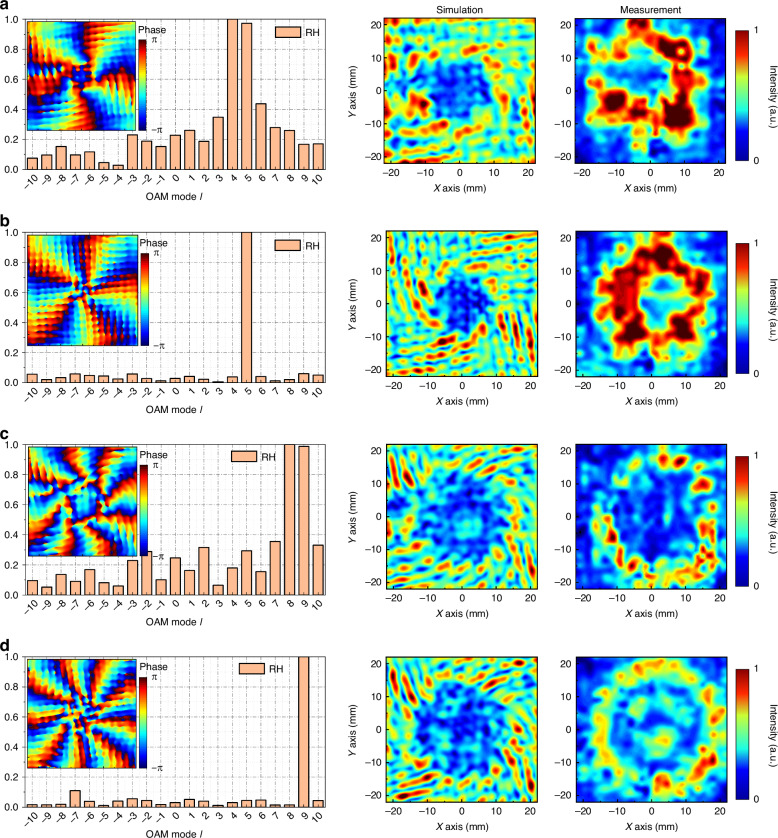
Schematic diagram of the near-field sampling results of various OAM transmission modes, including the simulated phase distribution, mode purity, simulated intensity distribution, and experimental intensity distribution. **a** *l* = 4.5 mode. **b** *l* = 5 mode. **c** *l* = 8.5 mode. **d** *l* = 9 mode

Figure 5 shows the near-field observation results of the four modules in the OAM transmission mode. The sampling planes of the simulation and experiment are both 10 mm away from the metamaterial. The incident beam is left-handed circularly polarized light. Figure 5a shows the effect of the *l* = 4.5 (MM1) module; the phase presents a counterclockwise spiral linear phase gradient distribution on the section, covering 4.5 phase periods. The mode purity analysis of the dextrorotatory component of the outgoing light shows that the weights of modes 4 and 5 are the highest, and the weights of the two modes are almost the same. The near-field light intensity distribution diagram shows that, except for the light intensity singularity in the center of the vortex light, the phase changes abruptly because of the existence of the fractional topological charges. The phase conflict results in a loss of light intensity, resulting in a notched donut-shaped distribution. Although the OAM phase and mode purity of the module are good, the output light intensity of the metamaterial module is not uniform, showing a discretized distribution, which is caused by the constraints of the unit on the incident light. The experimental results are similar to the simulation results. The circular light spot without devices is transformed into an open ring with a central cavity, which verifies the existence of the fractional vortex phase, and the light intensity distribution is also uneven. The data of MM2 are shown in Fig. 5b. Its phase distribution is uniform, and the weight of mode purity in mode 5 far exceeds that of the other modes. There is a singularity in the light intensity, and the experimental results match the simulation results. Under the same sampling distance, the light intensity singularities of MM1 and MM2 are similar in size, and MM1 has an extra light intensity gap, which exerts a certain effect on the light intensity after tight focusing. The near-field observation results of MM3 with higher-order topological charges are shown in Fig. 5c. Its phase covers 8.5 phase periods on the whole cross-section, and the weights of mode purity are concentrated on modes 8 and 9, which are equally weighted. Owing to the increase in the number of topological charges, at the same sampling distance, the central cavity of MM3 is larger than that of MM1, but it still presents a circular distribution of gaps. The experimental results meet the simulation expectations. Compared with the low topological charge mode, the discrete distribution of light intensity is more obvious. Figure 5d shows the near-field observation results of MM4. The integer-order phase distribution and mode purity are both good. The simulation and experimental light intensities are in line with the OAM characteristics. Compared with MM2, the cavity radius is significantly larger. All the modules are capable of outputting high-purity OAM beams.

**Fig. 6:**
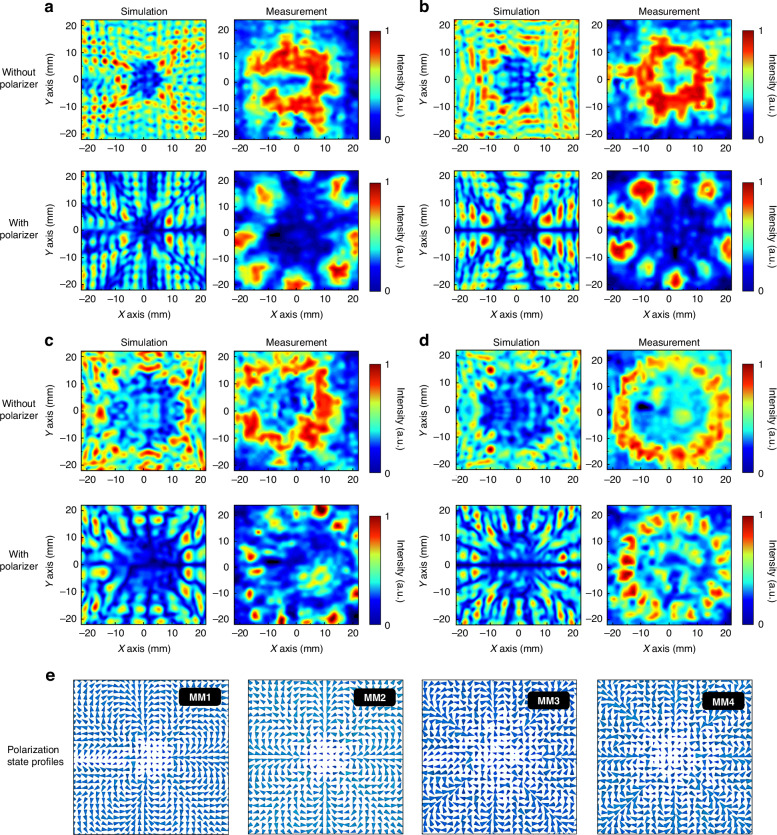
Schematic diagram of the sampling results of the OAM superimposed emission mode. **a** MM1 near-field sampling results. **b** MM2 near-field sampling results. **c** MM3 near-field sampling results. **d** MM4 near-field sampling results. **e** Distribution of the polarization states of modules MM1 to MM4

We tested the OAM superimposed emission mode of these four modules. The incident light was x-polarized light, and the sampling distance was also maintained at 10 mm when no polarization detection was performed. Since the linear polarizer has a certain size, the sampling distance of the polarization detection result is increased to 20 mm, as shown in Fig. 6. Figure 6a shows the near-field sampling results of MM1. The overall light intensity distribution also presents a discretized distribution, and there is still a phase singularity in the center. Compared with the OAM emission mode, there is also a certain loss of light intensity at the fractional phase superposition because of polarization conflicts. After x-polarization filtering is performed, the light intensity distribution is arranged in blocks with obvious boundaries. Except for the eight main light intensity intervals, the light intensity distributions are smaller than the complete phase interval at the fractional phase superposition. The near-field sampling results of the experiment verify this point. The light intensity distribution map contains a total of 8 strong light spots and one weak light spot. Owing to the increase in the sampling distance, the light intensity of the hole in the center increases. The test results of MM2 are shown in Fig. 6b. The distribution of the electric field intensity after the stacking of integer-order vortex beams is uniform, and the experimental intensity distribution is consistent with the simulation results. The simulated light intensity after passing through the linear polarizer presents 10 regions with clear boundaries, and the distributions of the 10 spots in the near-field experimental results are uniform, which matches well with the simulation results. The near-field test results of MM3 and MM4 are shown in Fig. 6c, d, respectively, which are similar to those of the low topological charge mode, and the simulation results are consistent with the experimental results. The discretization of the light intensity obscures the gap in the OAM superposition mode with high topological charge, but as shown in Fig. 6e, in the case where all *φ*_*0*_ values are 0, the polarization state distribution of each module conforms to the superposition result. The superposition effect of the integer order presents a lobe-like distribution, and the fractional order results in interference cancellation due to polarization conflicts at the junction.

**Fig. 7:**
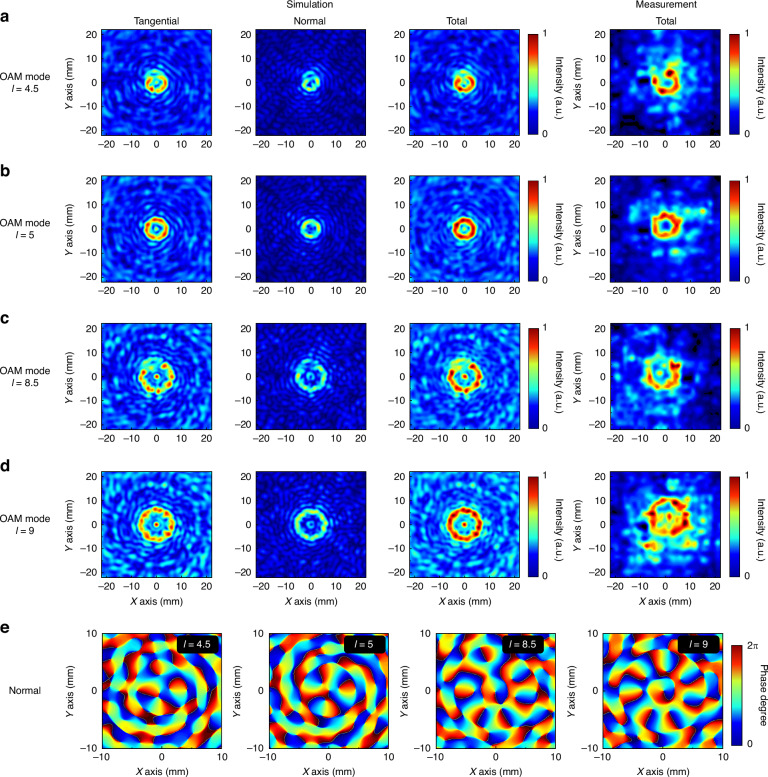
Schematic diagram of the simulation and experimental results of the proposed cascaded device, including the electric field strength and phase distribution. **a** Focal plane sampling analysis of the *l* = 4.5 mode. **b** Focal plane sampling analysis of the *l* = 5 mode. **c** Focal plane sampling analysis of the *l* = 8.5 mode. **d** Focal plane sampling analysis of the *l* = 9 mode. **e** Schematic diagram of the phase distribution of the longitudinal component in four different modes

Through experimental analysis of the metamaterial module, we found that the vector characteristics of all the modules in different emission modes are well maintained, but the light intensity is discretized because of the problem of array distribution density. Next, we can concatenate the light intensity by cascading the metamaterial module and the metalens module. A high numerical aperture lens (ML) can collect discrete light intensities and generate tightly focused fields in different vector states. The first is tight focusing research in the OAM emission mode. As shown in Fig. 7, the sampling distance of the simulation and the experiment is maintained at the focal point of 16 mm, whereas the left-handed circularly polarized light is still incident to facilitate comparison with the mode without a lens. The simulation near-field observation results after MM1 and ML are cascaded are shown in Fig. 7a. In our experiment, we utilized the Tera-1024 model camera, which features pixels measuring 32 × 32, with each pixel having a width of 1.5 mm. Consequently, the side length of the intensity distribution map obtained from our experimental testing is 48 mm. Using this side length as a reference, we employed a Vernier caliper to measure the spot diameter and then calculated the radius of the spot proportionally.

Owing to the irregularity of the edge of the spot and the finite sampling points of the camera, we conducted multiple measurements, evaluating the spot radius from different angles. Ultimately, we selected the average value as the measured spot radius. The overall light intensity presents an open ring distribution, and the lateral radius of the spot is 5 mm. The lens module converges well to the previously discrete light intensity and concentrates it at the focal point. The entire light intensity ring is evenly distributed. The existence of fractional phases results in obvious differences in light intensity. When the spatial coordinates of the whole field are decomposed, the tangential field component presents an open ring distribution similar to that of the total field, and its size is also consistent with the total field. The normal field presents an open ring distribution slightly smaller than the total field. The experimental results are consistent with the simulation results. The light intensity gap is obvious, and the edges of the rings are clear. The test results of MM2 are shown in Fig. 7b. Since there is no fractional phase, the overall light intensity presents a complete circular ring, the radius of the focal spot is ~5 mm, and there is a cavity with a radius of ~2.5 mm in the center. The distribution of light intensity in the ring is uniform. The tangential field component is also a ring with a radius of ~5 mm, but the width of the ring itself is smaller than the total field, and the normal field component is a ring with a radius of ~4.15 mm. The total field strength obtained from the experiment is consistent with the simulation results. Figure 7c shows the test results after cascading MM3. The introduction of higher topological charges significantly increases the size of the focusing aperture, and the gap also increases accordingly. The overall focal spot radius is 7.5 mm. Unlike in the low topological charge mode, a point-like focal point with a radius of 0.325 mm appears at the center of the singularity of the light intensity of the focal spot. The decomposed field components show that this focus also exists in the tangential field component, whereas the normal field component presents a double-ring distribution. The outer ring is a ring with a gap, and the center is a complete ring, while the light intensity of the central ring is smaller than that of the outer ring. The size of the gap ring and the central focus in the intensity distribution of the experimental test are consistent with those of the simulation. This is because the phase singularity of the OAM beam increases with the increasing number of topological charges added. However, the light intensity at the singular point of light intensity cannot always be kept at zero in reality, and after being collected by the lens, a tight focusing effect is also produced in the center of the spot. This phenomenon also occurs in the MM4 cascade test results shown in Fig. 7d. The total field halo radius is ~7.5 mm, and the center light spot radius is 0.325 mm. The experimental results are consistent with the simulation results. Figure 7e shows the phase distribution of the normal field component after all the modules are cascaded. We can see that after the OAM beam is tightly focused, its tangential component still maintains the original OAM mode number. The normal field generates a vector vortex beam with (*l* - 1) topological charge vortex phases, and its polarization direction is consistent with the wave vector direction. In the high topological charge mode, the center of the normal field produces a vector vortex light with first-order topological charge, and the topological charge of this beam does not change with the incident light OAM mode.

**Fig. 8:**
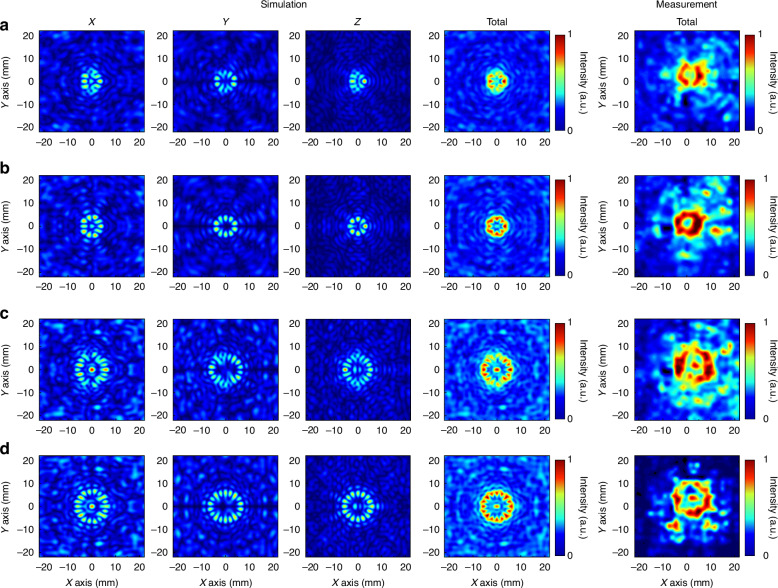
Properties of the proposed cascaded metamaterial capable of generating different OAM vortex beam superpositions with tightly focused fields. **a** MM1 focal plane sampling results. **b** MM2 focal plane sampling results. **c** MM3 focal plane sampling results. **d** MM4 focal plane sampling results

After completing the cascade analysis of different OAM vortex beams, the system switches to the conjugate OAM superposition mode. The incident light conditions are consistent with those in noncascade mode, and the near-field test results shown in Fig. 8 are obtained. The sampling plane was also placed at a focal length of 16 mm. The first is the cascaded output state of MM1 shown in Fig. 8a, where the root mean square of the x-component of the electric field presents a multifocal distribution. Owing to the existence of fractional superposition, the polarization conflict causes the focus light intensity at the top and bottom of the focal spot to be blurred, and the intensity is smaller than that at other positions. The focus position of the y component is orthogonal to the x component, and the light intensity is blurred at the same position as the x component. The z component intensity of the electric field generated by tight focusing is close to the x and y components, and the number of focal points is smaller than that of the other two components. There are also focal points with lower light intensities at the top and bottom. The superposition of the three causes the total field to present a double gap, and the light intensity distribution is in the shape of a bracket. The closer to the top and bottom, the weaker the light intensity. The focal spot radius is 4.5 mm. The experimental light intensity is consistent with the simulation results, and the size is slightly larger than the simulated spot. The results produced by MM2 after tight focusing are shown in Fig. 8b. The superposition of the integer-order vortex beams clearly produces a multifocal distribution of the three components. The number of focal points of the x and y components is the same; both are 10, and the spot radius is 5 mm. The number of focal points of the z component is eight, and the spot radius is smaller than that of the first two components, which is 4.5 mm. All the focal points have a uniform distribution of light intensity and the same size. The total field presents a complete ring, the spot radius is 5 mm, and the size is consistent with the OAM state. However, the light intensity distribution is not a uniform ring distribution under the OAM state, and the inner diameter presents a tooth-shaped distribution due to the existence of the z component. The focal spot radius produced by the experiment is 5 mm. Since the side length of the minimum pixel point of the camera is 1.5 mm and the distance between the multiple focus components is ~1.1 mm, the tooth-like distribution is not obvious, but the inner diameter is significantly smaller than that of the OAM state. Under the experimental results of the fractional-order OAM superposition state with high topological charge, the light intensity blur at the top and bottom of different components of the focal spot is more obvious, as shown in Fig. 8c. Compared with MM1, MM3 presents more focal points, and the intensities of the three components are similar. The spot radius of x and y is 7.5 mm, and the spot radius of z is 7 mm. The total field strength after superposition generally has a bracket-shaped distribution similar to that of MM1. Because of the multifocus distribution of the z component, the light intensity on both sides also presents a nonuniform distribution. The closer to the left and right vertices, the greater the light intensity, and the closer to the upper and lower vertices, the smaller the light intensity. As with the OAM emission mode, there is also a single small focal point in the center. The shape of the spot measured by the near-field test is consistent with the simulation results, and the radius of the spot is 8.5 mm, which is slightly larger than the simulation focal spot. Finally, the test results of MM4 are shown in Fig. 8d. Its x and y components generate 2*l* focal points with a spot radius of 8 mm, and the z components generate 2(*l*-1) focal points with a spot radius of 7 mm. The superposition of different radius components makes the total field appear smooth in terms of the outer diameter and multitoothed in terms of the inner diameter. The radii of the inner and outer diameters correspond to the radii of the components. The experimental results are also consistent with the simulation results, and the radius is 8.5 mm.

In the OAM emission mode, the fractional-order vortex beam can produce a notched ring-shaped spot, and the integer order is a complete ring. Both can produce the z component of the normal distribution of polarization, carrying the *1*-1 topological charge vortex phase, but the intensity of the z component is obviously smaller. After OAM superposition, the fractional-order light beam produces a bracket-shaped light intensity distribution with upper and lower double gaps, and the integer-order beam has a light intensity ring with a sawtooth-shaped inner diameter. Different polarization components present a multifocal distribution, and the intensity of the normal component z can be kept similar to that of other components. The metalens module converges well to the discrete light intensity of the metamaterial module. Under the multitopological charge mode and different emission modes, the overall experimental light spot can be well matched with the simulation in terms of light intensity distribution and size, which verifies the feasibility of the proposed scheme. The experimental and simulation results exhibit some degree of discrepancy, which can be attributed to the fact that the quality of the optical path itself can impact the test results during actual testing. The number of pixels in the camera we selected influences the final imaging effect. Insufficient sampling points can result in inadequate precision and detail with respect to the spot size of the light spot itself. It would be beneficial to replace the camera with higher imaging precision for the acquisition of test results, thereby enhancing the quality of the experimental results through optimized data collection.

During our numerical simulations, we were unable to match the intensity of the light source with the experimental beam data. Additionally, the output values from the terahertz camera we used do not represent the actual electric field intensity distribution, but rather the amplitudes output by the camera’s sensor units. Therefore, to ensure the reliability of the data in our quantitative analysis, we normalized both the simulation results and the experimental results.

The diffraction efficiency in this paper can be obtained by calculating the ratio of the power flow of the beam transformed by the all-dielectric metasurface to the power flow of the incident beam. The power flow is a far-field observable value, defined by the time-averaged radiant flux per unit area (the time-averaged magnitude of the Poynting vector) over configured distances: that is,

22 $${\boldsymbol{P}}= < {{\boldsymbol{E}}}_{far}\times {{\boldsymbol{H}}}_{far} >\, avg.$$

**Fig. 9:**
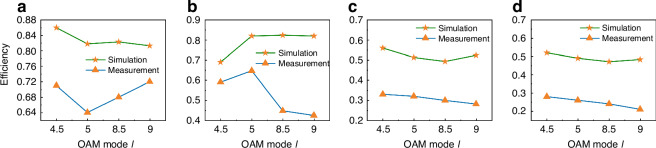
Comparison of simulation and experimental efficiency. **a** Simulation and experimental efficiency of different topological charge beams in the precascade conjugate superposition state. **b** Simulation and experimental efficiency of vortex beams with different topological charges in precascaded circularly polarized states. **c** Simulation and experimental efficiency of cascaded conjugate superposition states with different topological charge beams. **d** Simulation and experimental efficiency of cascaded circularly polarized vortex beams with different topological charges

On the basis of the above equation, we calculate the efficiency of different modules individually and in cascade and quantitatively compare the simulation and experimental results, as shown in Fig. 9.

The simulation and experimental efficiencies of the four modules before cascading are shown in Fig. 9a, b. Under the conjugate superposition state, the numerical simulation results show that the conversion efficiency of MM1 is 0.86, that of MM2 is 0.818, that of MM3 is 0.823, and that of MM4 is 0.813. The overall conversion efficiency decreases as the topological charge increases, but the difference is relatively small. The experimental results indicate that MM1 is 0.71, MM2 is 0.64, MM3 is 0.68, and MM4 is 0.72, with a small fluctuation range overall. Figure 9b shows the conversion efficiency of generating circularly polarized vortex beams via a single module. The simulation results show that MM1 is 0.69, MM2 is 0.82, MM3 is 0.824, and MM4 is 0.82, with little variation in the conversion efficiency compared with that of the conjugate superposition mode. The experimental results show that MM1 is 0.59, MM2 is 0.647, MM3 is 0.447, and MM4 is 0.424, indicating a greater decrease in the conversion efficiency compared to the conjugate superposition mode. The data during single-module testing are generally more scattered, and the stability between different topological charges is lower in the experiments than in the simulations.

The experimental and simulation data after cascading are shown in Fig. 9c, d. The stability of the focused light intensity clearly improved because of the constraints of the lens. Under the conjugate superposition state, the simulation conversion efficiency of MM1 is 0.56, that of MM2 is 0.513, that of MM3 is 0.493, and that of MM4 is 0.524, indicating a decrease in the overall device conversion efficiency due to cascading. The experimental results indicate that MM1 is 0.33, MM2 is 0.32, MM3 is 0.299, and MM4 is 0.281. The conversion efficiency in the circular polarization mode is close to that in the conjugate superposition state, and the trend remains consistent. The simulation results show that MM1 is 0.52, MM2 is 0.49, MM3 is 0.471, and MM4 is 0.483. Correspondingly, the experimental results show that MM1 is 0.28, MM2 is 0.26, MM3 is 0.24, and MM4 is 0.21.

## Conclusion

In summary, we demonstrate the use of 3D-printed all-dielectric cascade metamaterials to produce tight focal lengths of different vector beams. By making full use of the two degrees of freedom of propagation and the geometric phase, the characteristics of the tightly focused states of integer-order vortex beams, fractional-order vortex beams, and OAM superposition beams with conjugate topological charges are studied. By modulating the parameters of the rectangular column unit and the asymmetric bicolumn unit, the metamaterial module, and the metalens module were successfully constructed using low-refractive-index resin materials, and the metalens was physically cascaded with the metamaterial based on its flatness. Numerical simulations of the cascaded and noncascaded states were carried out on the designed module, and an optical path was built to characterize its propagation characteristics. Although there are small gaps between the simulated and ideal intensity distributions, these gaps can be improved by selecting more elements with better amplitude and phase characteristics. The cascading of the two modules compensates for the defect in the light intensity of the metamaterial module, and the experimental results are very consistent, which provides a new idea for further research on the tight focusing of different vector beams.
